# Disparities in satisfaction among insured and uninsured adult outpatient department service users in Southern Ethiopia, 2022: a comparative cross-sectional study

**DOI:** 10.1186/s12913-024-11176-9

**Published:** 2024-07-12

**Authors:** Mulugeta Hailu Rad, Tekle Ejajo, Legesse Tesfaye Elilo, Selamu Abose Nedamo, Dawit Sullamo, Aklilu Habte Hailegebireal, Belay Erchafo

**Affiliations:** 1 School of Public Health, College of Medicine and Health Sciences, Wachemo University, Hosanna, Ethiopia; 2 School of Nursing, College of Medicine and Health Science, Wachemo University, Hosanna, Ethiopia

**Keywords:** Patient satisfaction, Quality, Comparative, Insured, Uninsured, Ethiopia

## Abstract

**Background:**

Patient satisfaction is a critical measure of the quality of healthcare services provided by healthcare facilities. However, very few studies, particularly in Ethiopia, which includes the study area, have specifically examined these discrepancies among people who use outpatient care. In this study, satisfaction levels and associated factors were compared between insured and uninsured patients receiving outpatient services at public health institutions in Hadiya Zone, southern Ethiopia.

**Methods:**

A facility-based comparative cross-sectional study design was employed on 630 patients using multistage and systematic random sampling. Data were collected using a pretested and structured interviewer-administered questionnaire. Results of the analysis were presented in text, tables, and graphs as appropriate. Multivariable logistic regression was used to predict associations between predictors and the outcome variable. Statistical significance was declared at p-value < 0.05.

**Results:**

Overall, 344(55.48%) patients were satisfied with the service they received, of which 206(65.8%) out of 313 with a 95% CI [60.7–71.2%] were insured and 138(44.95%) out of 307 with a 95% CI [39.4–5.1%] were uninsured. Among insured patients, factors associated with higher satisfaction included having a family size less than five members [AOR = 3.3, 95% CI; 1.5, 7.4], perceived fair waiting time to be seen[AOR = 2.35, 95% CI; 1.02, 5.5], perceived short waiting time to be seen[AOR = 8.12, 95% CI; 1.6, 41.3], having all ordered laboratory tests available within the facility[AOR = 7.89, 95% CI; 3.5, 17.5], having some ordered laboratory tests within the facility[AOR = 2.97, 95% CI; 1.25, 7.01] having all prescribed medications available within the facility[AOR = 16.11, 95% CI; 6.25, 41.5], having some prescribed medications available within the facility[AOR = 13.11, 95% CI; 4.7, 36.4]. Among non-insured patients, factors associated with higher satisfaction included urban residency, a fair and short perceived time to be seen, having ordered laboratory tests within the facility, and having prescribed drugs within the facility.

**Conclusion:**

This study identified lower overall satisfaction, particularly among uninsured patients. Enrollment in the CBHI program significantly impacted satisfaction, with both groups reporting lower levels compared to enrollment periods. Access to essential services, wait times, and socio-demographic factors identified as associated factors with patient satisfaction regardless of insurance status.

**Supplementary Information:**

The online version contains supplementary material available at 10.1186/s12913-024-11176-9.

## Background

Patient satisfaction reflects the degree to which a patient’s expectations of healthcare providers align with the actual delivery of services. Numerous studies have identified factors influencing satisfaction, including aspects of the healthcare experience itself. A topic of particular interest is out-of-pocket expenses, which can significantly impact satisfaction, especially in developing countries, by limiting access to necessary healthcare services [1, 2].

Despite improvements in health service coverage since the global commitment to achieving Universal Health Coverage (UHC) by 2030, a significant portion of the population still lacks access to basic healthcare services. This lack of access often stems from high out-of-pocket costs incurred by patients seeking crucial medical care [3]. Recognizing this challenge, Ethiopia has implemented comprehensive financial risk protection programs along with health insurance schemes. Financial risk protection is a cornerstone of UHC. Notably, Community-Based Health Insurance (CBHI) covers a substantial portion of the population, exceeding 85%, particularly those in the informal sector [4]. The formal sector benefits from the Social Health Insurance (SHI) scheme, which is currently in its final stages of full implementation [4].

Several studies in Ethiopia have explored enrollment rates and overall satisfaction with the Community-Based Health Insurance (CBHI) program, reporting satisfaction prevalence ranging from 54 to 93.38%. These studies identified factors influencing satisfaction, including long waiting times, family size, availability of laboratory tests, medication availability, and healthcare provider friendliness [5–8]. Our research focused on patient satisfaction levels specifically among outpatients, categorized by their insured or uninsured status under CBHI. To address this, we conducted a study analyzing patient satisfaction with outpatient services at public health facilities in Hadiya Zone, Southern Ethiopia (details about the zone’s population, healthcare infrastructure, and CBHI scheme enrollment rate are provided in the Methods section). The enrollment rate for the CBHI program in Hadiya Zone (approximately 40% of households as per the zone health office’s 2020 CBHI report) presented a relevant setting to investigate satisfaction differences between insured and uninsured outpatients.

## Methods

### Study setting, period, and design

The study was conducted in Hadiya Zone, Southern Ethiopia. The zone’s capital city, Hossana, is located approximately 232 km south of Addis Ababa and 168 km south of Hawassa. Hadiya Zone has a population of 1,727,920 (according to a 2020 zone health department report) and includes various healthcare facilities, including hospitals, clinics, health centers, and health posts. Notably, the zone health office’s CBHI report indicated that approximately 40% of households were enrolled in the Community-Based Health Insurance (CBHI) scheme as of 2020. A comparative cross-sectional study design was employed for data collection during the period from July 15 to August 15, 2022.

### Population

#### Source population

The source populations were all patients who visited out-patient departments of public health facilities in the study period.

#### Study population

The study population included all adult patients who visited outpatient departments of public health facilities in the study area. These patients were either insured or non-insured under the CBHI scheme and were randomly selected.

Inclusion Criteria: The study included adult patients visiting outpatient departments of public health facilities during the study period, who were either insured or non-insured under the CBHI scheme.

#### Exclusion criteria

The study excluded the following.

- Formal sector employees.
- Clients utilizing exempted services.
- Critically ill patients.

### Sample size determination

We used Epi-Info 7 software to calculate the required sample size based on the following assumptions: 95% confidence interval, 80% power, and a 1:1 allocation ratio between insured and non-insured groups. We considered proportions of satisfaction from previous studies: 79% for insured and 66.5% for non-insured [9, 10]. A design effect of 1.5 and an anticipated non-response rate of 5% were factored in, resulting in a final sample size of 630.

### Sampling technique and procedure

We employed a multi-stage sampling technique, where the first step involved selecting 30% of the 13 woredas and one primary hospital. The total sample size of the study was proportionally allocated to randomly selected woredas. Then, 30% of the health centers from the selected woredas were selected by a simple random sampling technique. The calculated sample size was proportionally allocated to those selected health centers at selected worades based on the health centers’ OPD log books from the previous year (2021) of the same month. The lists of outpatient attendants were obtained from the registration books of the health facilities. During the study period, patients who visited the health facilities were registered as insured and non-insured in the record rooms using a daily routine register. Based on the list, interviewees were systematically selected from both insured and non-insured groups. The first study participant is selected using a lottery method from the register, and then a calculated k-value of approximately two is used to select the next (Table 1).

**Table 1:** Proportionally allocated sample size for selected woradas

Districts	# of patients in the last year similar month at selected health facilities		# patients allocated at selected facilities	Sample	
Anilemo	Total	338	156	uninsured	insured
	Fonko health center	208	96	48	48
	Achamo health center	130	60	30	30
Misha	Morsito health center	332	153	76	77
Amaka	Total	284	132	132	
	Geja health cener	193	89	44	45
	Gemedo health center	91	43	22	21
	Homacho primary Hospital	410	189	94	95
Total	1364			630	

### Variables and measurements

Patient satisfaction was dependent variable for both insured and uninsured.

Independent variables: Socio-demographic variables (patient’s age, sex, marital status, education status, occupation, income, family size, religion, residency), Health Service provision related factors and Health facility related factors.

Patient satisfaction was measured by sixteen items related to satisfaction in three dimensions. Staff behavior and services, physical facilities and environment, and accessibility and availability of health care services are asked on a five-point Likert scale, from strongly disagree” = 1 to strongly agree = 5. Then, the study participants were leveled as satisfied if their sum of responses was greater than or equal to the median value of the total sum score; otherwise, they were leveled as not satisfied [8].

### Data collection instruments and procedures

A structured questionnaire was developed for interviewer administration. The questionnaire was adapted from similar studies [2, 9, 11–14] to fit the context of this research. The final tool has three sections. The first section gathers background information, such as age, education level, and occupation. The second section focuses on factors related to health service provision for both insured and uninsured patients. The last section asks for participant satisfaction with 16 statements on a scale of 1 (strongly disagree) to 5 (strongly agree).

To ensure the questionnaire’s clarity across languages, the English version was translated to Amharic by one language expert and then back-translated to English by another. The Amharic version was then pretested at Lera Health Center in a neighboring district.

The reliability of the satisfaction measurement section was assessed using Cronbach’s alpha coefficient, which yielded a score of 0.83. This indicates good internal consistency for the measure. Finally, data collectors were deployed to the selected healthcare facilities to conduct data collection from study participants as they were exiting.

### Data quality management

Six BSc-holding nurses, fluent in Amharic and Hadiyisa, were selected as data collectors from health facilities outside of study facilities that participated in the study. Two public health officers provided close follow-up during data collection. All data collectors and supervisors received a one-day intensive training on data collection techniques, instruments, ethical considerations, and the study’s purpose. To ensure data quality, continuous supervision was provided by supervisors throughout the data collection period. Co-authors collaborated with the principal investigator to check the collected data for completeness, consistency, and clarity throughout this period. The data collection tool was pretested at Lera Health Center two days before data collection began on a sample of 32 participants (5% of the total sample). Based on the pretest results, any necessary corrections were made to the tool.

### Data processing and analysis

Data was entered into EpiData v3.1, exported to SPSS version 21, and cleaned to check for completeness and missing values. In descriptive statistics, frequencies, proportion, and mean were calculated. Results of the analysis were presented in text, tables, and graphs as appropriate. In bivariate analysis, variables with a p-value less than 25% were candidates for multivariable logistic regression analysis. Logistic regression analysis is used to identify factors associated with satisfaction among insured and non-insured patients. Before using the model, the goodness-of-fit, overall classification, and Pseudo R2 were all checked. At the end, adjusted odds ratios (AOR) were reported with a 95% confidence interval (CI) to measure the strength of association, and a p-value of 0.05 was used to determine statistical significance in all the statistical tests.

## Results

### Socio-demographic characteristics of the participants

A total of 620 participants responded to the study, yielding a remarkable response rate of 98.4%. The sample comprised 313 insured and 307 uninsured individuals. Both groups had an age range of 19–65 years, with insured patients averaging 37.04 years (SD ± 9.01) and uninsured patients averaging 37.77 years (SD ± 11.12). The majority were married (75.7% insured, 83.7% uninsured). Family size differed, with nearly two-thirds (67.4%) of insured patients having five or more family members compared to over half (53.4%) of uninsured patients having fewer than five. Literacy also differed, with 51.1% of insured and 44.0% of uninsured patients unable to read and write. Farming was the most common occupation (43.5% insured, 50.8% uninsured). Residence also varied, with more than half residing in rural areas (62.9% insured, 54.7% uninsured). The majority of respondents identified as Protestant religious followers (Table 2).

**Table 2:** Socio-demographic characteristics of the respondents at outpatient departments of Hadiya zone primary health care unit 2022

Variable		Insured (*N* = 313) *n* (%)	Non-insured(*N* = 313) *n* (%)	Total(*N* = 620) *n*(%)	Test statistics
Sex (*N* = 620	Male	180(57.5%)	168(54.7%)	348(56.1%)	χ^2^ = 0.488 *p* = 0.85
	Female	133(42.5%	139(45.3%	272(43.9%)	
Marital status(*N* **=** 620)	Married to one spouse	249(79.6%)	275(89.6%)	524(84.5%)	χ^2 =^ 12.258 *p* = 0.002
	Married to > 1 spouse	44(14.1%)	24(7.8%)	68(11.0%)	
	Not in marriage	20(6.4%)	8(2.6%)	28(4.5%)	
Family size(*N* = 620)	< 5	102(32.6%)	164(53.4%)	266(42.9%)	χ^2 =^ 27.458 *p* < 0.001
	≥ 5	211(67.4%)	143(46.6%)	354(57.1%)	
Educational status (*N* = 620)	Unable to read and write	160(51.1%)	135(44.0%)	295(47.6%)	χ^2 =^ 8.430 *p* = 0.015
	Elementary school (1–8)	119(38.0%)	114(37.1%)	233(37.6%)	
	Above 9	34(10.9%)	58(18.9%)	92(14.8%)	
occupation	Farmer	136(43.5%)	156(50.8%)	292(47.1%)	χ^2 =^ 3.903 *p* = 0.14
	Private	109(34.8%)	87(28.3%)	196(31.6%)	
	Unemployed	68(21.7%)	64(20.8%)	132(21.3%)	
	Protestant	256(81.8%)	216(70.4%)	472(76.1%)	χ^2 =^ 16.33 *p* = 0.12
Religion	Orthodox	39(12.5%)	69(22.5%)	108(17.4%)	
	Muslim	14(4.5%)	10(3.3%)	24(3.9%)	
	other	4(1.3%)	12(3.9%)	16(2.6%)	
Residency	urban	116(37.1%)	139(45.3%)	255(41.1%)	χ^2 =^ 4.321 *p* = 0.03
	rural	197(62.9%)	168(54.7%)	365(58.9%)	
Age		37.04 ± 9.01 years	37.77 ± 11.12 years		*P* = 0.37

### Interaction and health service related factors

This study revealed a high rate of patient identification verification before service provision, with 74.4% of insured and 69.4% of uninsured patients reporting confirmation of their identity (*n* = 233 and *n* = 213, respectively). This finding suggests a generally good practice in ensuring patient safety and accurate medical record association across both insurance groups. However, the data highlights a significant disparity in the reported comprehensiveness of physical examinations. Over 40% of insured patients reported receiving a thorough examination, while only around 25% of uninsured patients did (Table 3).

**Table 3:** Interaction and Health Service provision related factors for patients at OPD of Hadiya Zone Health Facilities (*n* = 620)

Variable		Insured	Non-insured	Total	Test statistics
interviewed by the language they can understand	Yes	135(43.1%)	142(46.3%)	277(44.7%)	χ^2 =^ 0.61 *p* = 0.43
	No	178(56.9%)	165(53.7%)	343(55.3%)	
Patients identity is confirmed by service provider	Yes	233(74.4%)	213(69.4%)	446(71.9%)	χ^2 =^ 0.19 *p* = 0.16
	No	80(25.6%)	94(30.6%)	174(28.1%)	
Did the provider examine the patient (head to toe)	Yes	127(40.6%)	78(25.4%)	205(33.1%)	χ^2 =^ 16.11 *p* < 0.001
	No	186(59.4%)	229(74.6%)	415(66.9%)	
history of past illness	Yes	148(47.3%)	200(65.1%)	348(56.1%)	χ^2 =^ 20.08 *p* < 0.001
	No	165(52.7%)	107(34.9%)	272(43.9%)	
history of present illness	Yes	306(97.8%)	295(96.1%)	601(96.9%)	χ^2 =^ 1.459 *p* = 0.16
	No	7(2.2%)	12(3.9%)	19(3.1%)	
treatment was taken before arrival at facility	Yes	217(69.3%)	169(55.0%)	386(62.3%)	χ^2 =^ 13 *p* = 0.48
	No	96(30.7%)	138(45.0%)	234(37.7%)	
provider explain the diagnosis to the patient	Yes	211(67.4%)	165(53.7%)	376(60.6%)	χ^2 =^ 13 *p* = 0.48
	No	102(32.6%)	142(46.3%)	244(39.4%)	

### Institutional aspect and pattern of visit

A total of 408 or 65.8% of patients reported it was their first visit 187(59.7%) for insured and 221(72.0%) non- insured. With regard to perceived waiting time to see physician 360 or 58.1(229 or 73.2% insured and 131(42.7%) non-insured patients reported it was fair. A total of 538(86.8%) patients get laboratory order. From those lab ordered 269(50%) did get all the investigations. One hundred forty seven (51.2%) from insured and 122(48.6%) from uninsured did get all tests within the facilities (Table 4).

**Table 4:** institutional aspect and pattern of visit at Hadiya Zone health facilities OPD (*n* = 620)

Variable		Insured	Non-insured	Total	Test statistics
Number of visit within the last 24 months	One time	187(59.7%)	221(72.0%)	408(65.8%)	χ2 = 10.436 *p* = 0.012
	Two times	104(33.2%)	72(23.5%)	176(28.4%)	
	Three times	18(5.8%)	12(3.9%)	30(4.8%)	
	Four times	4(1.3%)	2(0.7%)	6(1.0%)	
Perceived waiting time to see physician	Long	58(18.5%)	139(45.3%)	197(31.8%)	χ2 = 61.851 *p* < 0.001
	Fair	229(73.2%)	131(42.7%)	360(58.1%)	
	Short	26(8.3%)	37(12.1%)	63(10.2)	
Perceived consultation duration	Long	135(43.1%)	83(27.0%)	218(35.2%)	χ2 = 18.48 *p* < 0.001
	Fair	112(35.8%)	151(49.2%)	263(42.4%)	
	Short	66(0.1%)	73(23.8)	139(22.4%)	
Laboratory test ordered(*N* = 620)	Yes	287(91.7%)	251(81.8%)	538(86.8%)	χ2 = 13.32 *p* < 0.001
	No	26(8.3%)	56(18.2%)	82(13.2%)	
Availability of ordered laboratory in facility(*N* = 538)	All available	147(51.2%)	122(48.6%)	269(50%)	χ^2 =^ 1.279 *p* = 0.52
	Some available	70(24.4%)	57(22.7%)	127(23.6%)	
	None available	70(24.4%	72(28.7%)	142(26.4)	
Availability of prescribed drugs(*N* = 620)	All available	138(44.1%)	111(36.2%)	249(40.2%)	χ^2 =^ 16.75 *p* < 0.001
	Some available	96(30.7%)	72(23.5%)	168(27.1%)	
	None available	79(25.2%)	124(40.4%)	203(32.7%)	
Information gained on drug use and side effects(*N* = 539)	Explain all	109(46.6%)	80(43.7%)	189(45.3%)	χ^2^_=_0 0.342 *p* = 0.83
	Explain some	70(29.9%)	58(31.7%)	128(30.7%)	
	Do not explain	55(23.5%)	45(24.6%)	100(24.0%)	
Satisfaction due to the cost of services	Satisfied	245(78.3%)	89(29.0%)	334(53.9%)	χ^2 =^ 151.9 *p* < 0.001
	Not satisfied	68(21.7%)	218(71.0%)	286(46.1%)	

### Patient satisfaction

In this study with regard to staff behavior 40(13%) for uninsured and 92 (29%) patients agreed they being treated friendly, 81(26%) for insured and 92(29%) for insured disagreed they did get explanation how to prevent the disease, 69(22%) uninsured and 74(23%) insured patients disagreed the professionals are careful when they examining and treating. When we see about environment 75(24%) from uninsured and 69(22%) from insured agreed the location of OPD is convenient, 82(26%) from uninsured 92(29%) disagreed waiting area chairs are confortable. About accessibility and availability of health services 55(17%) from uninsured and 123(39%) from agreed that they are satisfied with overall waiting time (Table 5).

**Table 5:** Level of satisfaction of patients on each satisfaction measuring items for non-insured at OPD Hadiya Zone Health Facilities (*n* = 620)

Items	CBHI status	Strongly Disagree *n*(%)	Disagree *n*(%)	Neutral *n*(%)	Agree *n*(%)	Strongly Agree *n*(%)
Health providers treats you very friendly and courteous manner	Uninsured	7(2.3)	110(35.8)	146(47.6)	40(13.0)	4(1.3)
	Insured	19(6.1)	86(7.5)	88(28.1)	92(29.4)	28(8.9)
Health providers are good to explain how to prevent your disease	Uninsured	4(1.3)	81(26.38)	117(38.1)	75(24.42)	30(9.7)
	Insured	0	92(29.4)	121(38.7)	82(26.2)	18(5.8)
Health providers are careful to check everything when treating and examining me	Uninsured	7(2.28)	69(22.4)	110(35.8)	97(31.6)	24(7.8)
	Insured	10(3.2)	74(23.6)	124(39.6)	93(30.2)	12(3.4)
You are satisfied with the information provided by health providers (courteous and respectful)	Uninsured	9(2.9)	112(36.5)	95(30.9)	71(23.1)	20(6.5)
	Insured	4(1.3)	68(21.7)	116(37.1)	115(36.7)	10(3.2)
You are satisfied with the information provided by all other staffs (other than health providers)	Uninsured	0	110(35.8)	139(45.3)	50(16.3)	8(2.6)
	Insured	8(2.6)	117(37.4)	120(38.3)	52(16.6)	16(5.1)
You are satisfied with the way health providers listened to you	Uninsured	3(1.0)	111(36.2)	104(33.9)	59(19.2)	30(9.8)
	Insured	16(5.1)	64(20.4)	135(43.1)	82(26.2)	16(5.1)
You are satisfied with measures taken to assure your confidentiality	Uninsured	5(1.6)	130(42.3)	105(34.2)	59(19.2)	8(2.6)
	Insured	20(6.4)	91(29.1)	95(30.4)	83(26.5)	24(7.7)
You are satisfied with the overall quality of health care services in this health facility	Uninsured	10(3.3)	37(12.1)	126(41.0)	112(36.5	22(7.2)
	Insured	4(1.3)	66(21.1)	125(39.9)	84(26.8)	34(10.9)
Adult OPD location is convenient for you	Uninsured	25(8.1)	72(23.5)	63(20.5)	75(24.4)	72(23.5)
	Insured	20(6.4)	69(22.0)	121(38.7)	69(22.4)	34(10.8)
The chairs in the waiting area were confortable	Uninsured	38(12.4)	82(26.7)	98(31.9)	83(27.0)	6(2.0)
	Insured	12(3.8)	92(29.4)	94(30.0)	97(31.0)	18(5.8)
Waiting area was clean	Uninsured	32(10.4)	74(24.1)	133(43.3)	54(17.6)	14(4.6)
	Insured	13(4.2)	73(23.3)	109(34.8)	102(32.6)	16(5.1)
Examination/consultation room/Outpatient department was clean	Uninsured	24(7.8)	48(15.6)	104(33.9)	105(34.2)	26(8.5)
	Insured	20(6.4)	38(12.1)	125(39.9)	80(25.6)	50(16.0)
Overall the compound is clean	Uninsured	60(19.5)	101(32.9)	85(27.7)	53(17.3)	8(2.6)
	Insured	16(5.1)	107(34.2)	104(33.2)	70(22.4)	16(5.1)
Time to get outpatient services after registration (at Waiting area) appropriateness for you	Uninsured	39(12.7)	93(30.3)	54(17.6)	95(30.9)	26(8.5)
	Insured	18(5.8)	75(24.0)	102(32.6)	76(24.3)	42(13.4)
The time spent to get services and get back (overall waiting time)	Uninsured	23(7.5)	98(31.9)	107(34.9)	55(17.9)	24(7.8)
	Insured	8(2.6)	76(24.3)	92(29.4)	123(39.3)	14(4.5)
Satisfied with the consultation duration	Uninsured	22(7.2)	110(35.8)	100(32.6)	57(18.6)	18(5.9)
	Insured	36(11.5)	69(22.0)	120(38.3)	58(18.5)	30(9.6)

### Satisfaction of patients and CBHI status

**Fig. 1:**
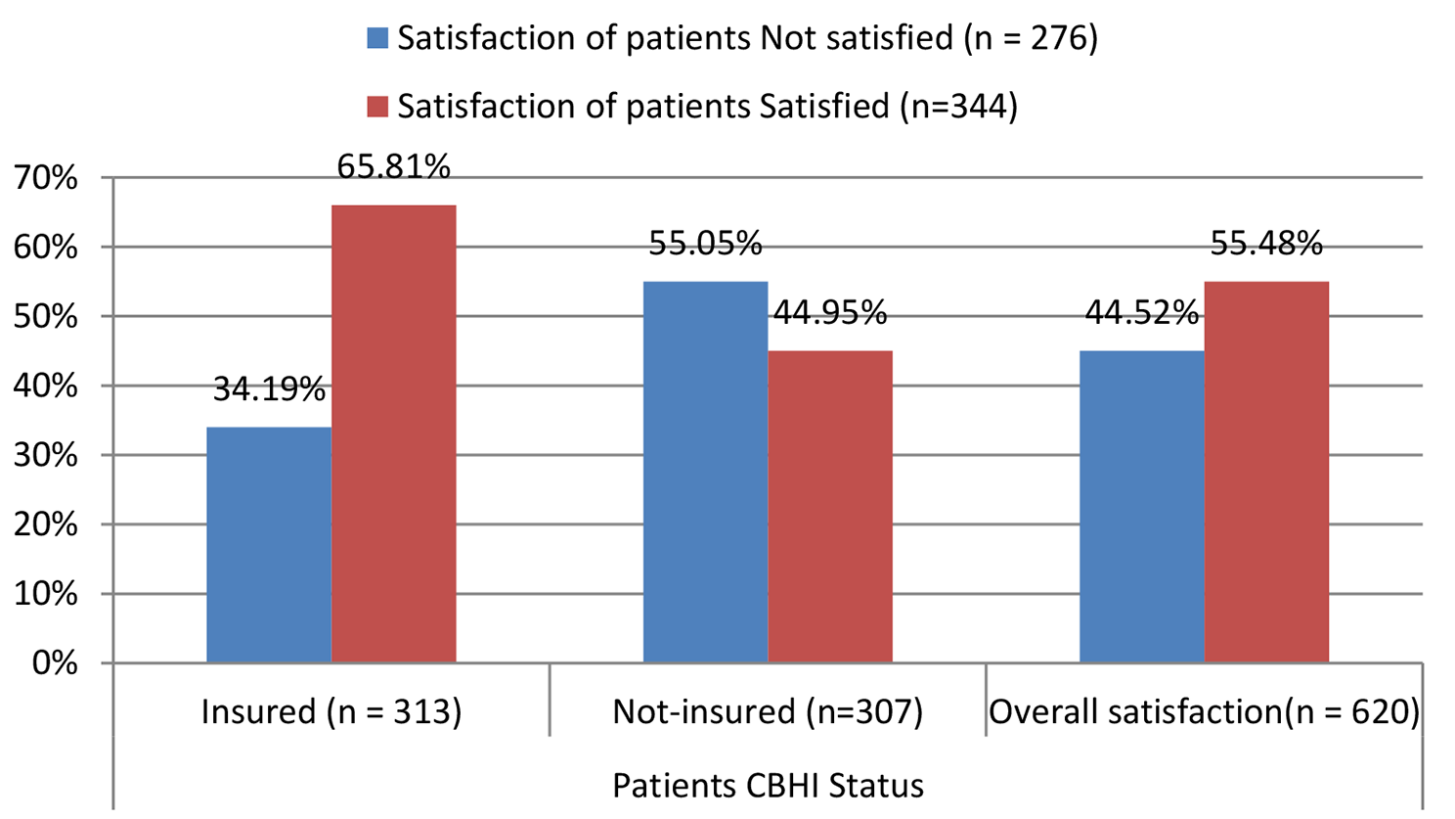
Satisfaction of patients * patients CBHI status

In general, more than half of the patients (344, or 55.48%) were satisfied with the health services. When we look at CBHI status with satisfaction of patients, the majority of them were from those who had CBHI membership; 206 (65.81%) with a 95% CI [60.7–71.2%]. In order to check whether those differences are statistically significant, chi-square testing was done (χ2 = 27.3, *p* < 0.001) (Fig. 1).

### Factors associated with satisfaction of patients

In the multivariable logistic regression analysis, five of the eight candidate variables were statistically associated with patient satisfaction. Patients in the CBHI scheme or who were insured were nearly twice as likely to be satisfied as non-insured patients; their AOR = 1.84 (1.13, 3.02). When compared to their counterparts, urban residents were twice as likely to be satisfied. AOR = 2.35 (1.41, 3.92). Patients who believe the wait time to see a physician is fair or short are four times more likely to be satisfied than those who believe it is long. AOR = 4.17(2.427, 7.17) and AOR = 4.47(2.03, 9.87). Availability of ordered laboratories within the facility: when they get all of them eleven times, some of the ordered laboratories are three times more likely to be satisfied compared with those who did not get ordered laboratories within the facility. AOR = 11.78 (6.50, 21.33), and 3.44 (1.82, 6.51). Patients are twelve times more likely to be satisfied when they receive all of the prescribed drugs; when they receive some of the prescribed drugs, they are five times more likely to be satisfied when compared to receiving none. AOR = 12.53 (6.80, 23.06) and AOR = 5.19 (2.82, 9.57), respectively (Table 6).

**Table 6:** Binary and multi-variable logistic regression analysis to identify factors associated with satisfaction of patients at Hadiya Zone Health Facilities (*n* = 620)

Independent variables		Not satisfied	Satisfied	at binary level		Multi-variable level AOR(95% CI)	
				*p*- value	COR(95%CI)		*p*-value
Sex	Male	146	202	0.147	1.2(0.92, 1.74)	0.93(0.57, 1.54	0.80
	Female	130	142		1	1	1
Marriage status	Married to one spouse	247	277	0.002	0.18(0.06, 0.5)	0.91(0.25, 3.24)	0.34
	Married to more than one spouse	25	43	0.036	0.2(0.08,0.92)	1.69(0.37, 7.640)	0.88
	Not in marriage	4	24	0.003	1	1	0.49
Residency	urban	92	163	< 0.001	1.8(1.29, 2.5)	2.35(1.41, 3.92)*	0.001
	rural	184	181	1	1	1	1
Occupation	Farmer	138	154	0.015	1	1	0.64
	Private	94	102	0.88	0.9(0.67, 1.39)	0.72(0.37, 1.41)	0.34
	Unemployed	44	88	0.008	1.7(1.16, 2.7)	0.81(0.39, 1.70)	0.58
CBH status of patients	Insured	107	206	< 0.001	2.3(1.7, 3.26)	1.84(1.13, 3.02)*	0.014
	Non-insured	169	138	1	1	1	1
Perceived time to see physician	Long	129	68	< 0.001	1	1	< 0.001
	Fair	125	235	< 0.001	3.5(2.4, 5.13)	4.17(2.427, 7.17)*	< 0.001
	Short	22	41	< 0.001	3.5(1.9, 6.41)	4.47(2.03, 9.87)*	0.03
Availability of ordered laboratory in facility	All available	66	203	< 0.001	7.8(4.95, 2.4)	11.78(6.50, 21.33)*	< 0.001
	Some available	60	67	< 0.001	2.8(1.71, 4.71)	3.44(1.82, 6.51)*	< 0.001
	None available	102	40	< 0.001	1	1	< 0.001
Availability of prescribed drugs	All available	71	208	< 0.001	12.3(7.8, 19.2)	12.53(6.80, 23.06)*	< 0.001
	Some available	65	103	< 0.001	5.4(3.4, 8.5)	5.19(2.82, 9.57)*	. <0.001
	None available	140	33	< 0.001	1	1	< 0.001

1 = reference group, * = statistically significant at p-value < 5%

Model fitness checking parameters: **Hosmer and Lemeshow Test** = 0.35, **Nagelkerke R Square** 0.655 and **Classification Table** Overall Percentage = 87.0. All the parameters are more than expectation. So, the model is good enough to use

A multivariable logistic regression analysis of insured patients identified four out of six candidate variables associated with patient satisfaction. Family size played a role, with those having less than five members being three times more likely to report satisfaction compared to those with larger families (AOR = 3.30, 95% CI: 1.50, 7.40). Waiting time significantly impacted satisfaction. Participants perceiving a fair waiting time were twice as likely to be satisfied compared to those experiencing a long wait (AOR = 2.35, 95% CI: 1.02, 5.50). Interestingly, perceiving a short waiting time had an even stronger association, with participants eight times more likely to be satisfied compared to those with a long wait (AOR = 8.12, 95% CI: 1.60, 41.30). One of the strongest contributors to patient satisfaction among insured patients was the availability of laboratory tests within the facility. Having all ordered tests available was associated with nearly eight times the odds of satisfaction compared to none being available (AOR = 7.89, 95% CI: 3.50, 17.50). Similarly, having some tests available increased satisfaction, with participants three times more likely to be satisfied compared to having no tests available (AOR = 2.97, 95% CI: 1.25, 7.01) (Table 7).

**Table 7:** Binary and multi-variable logistic regression analysis to identify factors associated with satisfaction of insured patients at Hadiya Zone Health Facilities (*n* = 313)

Independent variables		Not satisfied	Satisfied	at binary level		Multi-variable level	
				*p*- value	COR(95%CI)	AOR(95% CI)	*p*-value
Marriage status	Married to one spouse	95	154	0.12	0.4(0.132 1.248)	1.2(0.28, 4.9)	0.80
	Married to more than one spouse	8	36	0.86	1.1(0.295 4.283)	1.62 (0.29, 9.1)	0.50
	Not in marriage	4	16	0.017	1	1	072
Family size	< 5	22	80	0.001	2.45(1.4,4.2)	3.35(1.5, 7.4)*	0.003
	*≥* 5	85	126	1	1	1	1
Occupation	Farmer	50	86	0.059	0.53(0.27, 1.024)	0.58(0.2, 1.6)	0.28
	Private	41	68	0.053	0.51(0.26, 1.009)	0.56 (0.2, 1.6)	0.26
	Unemployed	16	52	0.11	1	1	0.50
Perceived time to see physician	Long	24	34	0.25	1	1	0.021
	Fair	77	152	0.27	1.4(0.77, 2.5)	2.35(1.02, 5.5)*	0.031
	Short	6	20	0.11	2.4(0.82, 6.5)	8.12 (1.6, 41.3)*	0.012
Availability of ordered laboratory in facility	All available	23	124	< 0.001	7.6(3.9, 14.6)	7.89(3.5, 17.5)*	< 0.001
	Some available	27	43	0.019	2.6(1.2, 4.4)	2.97 (1.25, 7.01)*	0.012
	None available	41	29	< 0.001	1	1	< 0.001
Availability of prescribed drugs	All available	35	123	0.001	13.7(6.5, 28.7)	16.11(6.25, 41.5)*	< 0.001
	Some available	25	71	0.001	11.1(5.1, 24.2)	13.11 (4.7, 36.4)*	< 0.001
	None available	47	12	< 0.001	1	1	< 0.001

Key: 1: Reference category; *AOR* Adjusted odds ratio, *COR* Crude odds ratio, * statistically significant at *p*-value < 0.05

Model fitness checking parameters: **Hosmer and lemshow** *p* = 0.39, **Nagelkerke R Square** = 0.45 and **Classification Table** Overall Percentage = 82.2

A multivariable logistic regression analysis of non-insured patients identified five out of seven candidate variables associated with patient satisfaction. Living in an urban area was associated with six times the odds of satisfaction compared to their counterparts [AOR = 6.38, 95% CI; 2.54, 16.03]. Similarly, perceiving a fair waiting time was associated with eight times higher odds of satisfaction compared to those who perceived a long wait [AOR = 8.55, 95% CI: 3.22, 22.67]. The availability of laboratory tests within the facility also played a significant role. Having all ordered tests available was associated with 19 times higher odds of satisfaction compared to none being available [AOR = 19.58, 95% CI; 6.43, 59.6]. Even having some tests available increased satisfaction, with participants four times more likely to be satisfied compared to having no tests available [AOR = 4.57, 95% CI; 1.33, 15.73] (Table 8).

**Table 8:** Binary and multi-variable logistic regression analysis to identify factors associated with satisfaction of non-insured patients at Hadiya Zone Health Facilities (*n* = 307)

Independent variables		Not satisfied	Satisfied	at binary level		Multi-variable level	
				*p*- value	COR(95%CI)	AOR(95% CI)	*p*-value
educational status	Unable to read and write	78	57	0.007	0.42 (0.2, 0.78)	0.45(0.14, 1.423)	0.12
	Grade 1–8	70	44	0.002	0.34(0.1, 0.69)	0.41(0.12, 1.44)	0.18
	Grade 9 and above	21	37	0.06	1	1	0.27
Occupation	Farmer	88	68	0.089	0.6(0.3, 1.080)	1.65(0.50, 5.40)	0.78
	Private	53	34	0.038	0.49(0.26, 0.96)	4.26(0.91, 19.8)	0.15
	Unemployed	28	36	0.10	1	1	0.22
Residency	Urban	55	84	0.001	3.22,(2.01, 5.15)	6.38(2.54, 16.03)*	< 0.001
	rural	114	54	0.001	1	1	1
Number of frequency	One time	132	89	0.027	1	1	0.36
	Two times	34	38	0.064	1.65(0.97, 2.83)	3.10(1.03, 9.30)*	0.07
	Three times	2	10	0.011	7.41(1.5, 34.65)	1.44(0.188, 11.09)	0.65
	Four time or more	1	1	0.781	1.48(0.0, 24.02)	1.66(0.001, 3071.79)	0.88
Perceived time to see physician	Long	105	34	0.001	1	1	< 0.001
	Fair	48	83	0.001	5.3(3.15, 9.03)	8.55(3.22, 22.67)*	< 0.001
	Short	16	21	0.001	4.05(1.9, 8.63)	2.63(0.785, 8.82)	0.098
Availability of ordered laboratory in facility	All available	43	79	< 0.001	10.18(4.85, 21.32)	19.58(6.43, 59.6)*	< 0.001
	Some available	33	24	< 0.001	4.03(1.76, 9.25)	4.57(1.33 15.73)*	0.01
	None available	61	11	0.001	1	1	< 0.001
Availability of prescribed drugs	All available	36	85	< 0.001	10.45(5.6, 19.3)	27.46(8.89, 84.79)*	< 0.001
	Some available	40	32	< 0.001	3.54(1.82, 6.88)	3.94(1.13, 13.63)*	0.031
	None available	93	21	< 0.001	1	1	< 0.001

Key: 1: Reference category; *AOR* Adjusted odds ratio, *COR* Crude odds ratio, * statistically significant at *p*-value < 0.05

Model fitness checking parameters: **Hosmer and lemshow** *p* = 0.24, **Nagelkerke R Square** = 0.68 and **Classification Table** Overall Percentage = 85.7

## Discussion

One of the indicators of quality health care services is the patient’s perspective. Patient satisfaction is a proxy indicator for the quality of services. This study compared patient satisfaction with OPD services under a CBHI program in southern Ethiopia. The findings revealed that over half of the patients (55%) expressed satisfaction with the services they received. However, a significant disparity occurred between insured and non-insured patients. Insured patients reported considerably higher satisfaction (66%) compared to their non-insured counterparts (45%). This difference suggests that health insurance status plays a crucial role in shaping patient satisfaction with OPD services. In identifying the reasons behind this difference and other factors influencing satisfaction, the study identified several key associations. These include factors like family size, residency status, perceived waiting times, availability of laboratory tests within the facility, and having prescribed medications within the facility.

Overall patient satisfaction in this study was 55%. aligns with a Gondar study that reported 56.1% (95% CI: 51.0–61.3)) [13]. However, it falls short of findings from studies conducted in Gurage Zone primary hospitals (66.5%) [10], Addis Ababa public hospitals (89.3%) [14], and Secondary Care Hospital in Northern India reported high satisfaction, with 77% rating services above 3.8 out of 5 [15]. These differences might be due to variations in facility types. The studies with higher satisfaction rates were conducted in more urban settings, where patients likely have better access to essential medical supplies and transportation. Disaggregating the data by insurance status revealed a significant disparity between insured (66%) and non-insured patients (45%) in our study. Insured patients were nearly twice as likely to be satisfied. This finding aligns with research from northern Ethiopia and a northern Nigerian hospital, where a similar association between insurance status and satisfaction was observed. However, the satisfaction gap between insured and non-insured patients in this study is wider compared to the findings from these previous studies (79.4% vs. 75.7% in northern Ethiopia [9] and 94% vs. 93% in northern Nigeria [16]. This difference might be due to the time frame of the studies. Previously, accessing healthcare at government facilities in Ethiopia was more affordable. However, recent national inflation has increased the cost of drugs and reagents, making it more difficult for patients to obtain necessary services. Furthermore, there is a scarcity of drugs and reagents within the facilities themselves. Even if an un-insured patient could afford the medication, it might not be available, further reducing their satisfaction with the service. In contrast, insured patients are typically reimbursed for the cost of medications if they are available. While the availability of medications can still be an issue, having insurance provides a layer of security regarding affordability. This financial security likely contributes to the higher satisfaction levels reported by insured patients in this study, despite these levels being lower than those reported in earlier studies.

Furthermore, our study found that family size was associated with patient satisfaction among insured patients, with smaller families reporting higher satisfaction levels. Several potential explanations exist for this association within the insured population. For example, insured patients with smaller families might experience less financial stress regarding potential co-pays or uncovered expenses compared to those with larger families.

Disaggregating the data by insurance status revealed that residence played a significant role in patient satisfaction among the non-insured population. Patients residing in urban areas reported higher satisfaction levels compared to their rural counterparts. Interestingly, our study found a different pattern compared to previous research conducted at Dilla Hospital, which reported no significant difference in satisfaction based on residence [17]. This discrepancy may be attributable to the challenges faced by non-insured patients from rural areas who must travel long distances to access healthcare facilities. The associated fatigue, frustration, and potential financial strain related to transportation costs could all contribute to lower satisfaction with services.

Furthermore, as expected, perceived waiting time to see a physician identified occurred as a significant factor influencing patient satisfaction in our study. Patients who reported fair or short waiting times expressed higher satisfaction compared to those who perceived a long wait. This finding aligns with research conducted in Addis Ababa, Ethiopia, which demonstrated a negative impact of long wait times on patient satisfaction with OPD services [14]. Similar associations have been observed in other settings, such as Nepal and Southern Rwanda [18, 19], highlighting the importance of efficient service delivery in outpatient care.

In line with previous research emphasizing the importance of resource availability [5, 14], our study revealed a significant association between patient satisfaction and the availability of essential resources within the healthcare facility. This association held true for both laboratory tests and prescribed medications. Patients who reported having at least some of their ordered laboratory tests or prescribed medications available within the facility expressed higher satisfaction compared to those who had none available. Interestingly, this association appeared to be more pronounced for non-insured patients after disaggregating the data by insurance status. While the sample size for each group decreased upon disaggregation, the results suggest that the unavailability of essential resources within the facility might have a particularly strong negative impact on the satisfaction of non-insured patients.

Several reasons might explain why resource availability has a more prominent impact on non-insured patients’ satisfaction. Previous research has documented the negative consequences of medication and laboratory test non-availability on patient satisfaction [14]. In the case of non-insured patients, the unavailability of these resources within the facility can lead to additional burdens: In contrast, the impact on insured patients in this context is likely mitigated to some extent by their insurance coverage. However, it’s important to consider the specific limitations of insurance coverage in our country. Here, insurance typically only covers laboratory services and medications performed within government healthcare facilities. Therefore, even insured patients who need to utilize external facilities (often private facilities) would face additional out-of-pocket costs not covered by insurance. While these costs might be lower compared to those faced by entirely non-insured patients, they can still contribute to reduced satisfaction.

This study revealed that patient satisfaction with OPD services under the CBHI program was considerably lower than that reported in previous studies [5, 7–9, 14], with a significant disparity between insured and non-insured patients. These findings highlight the need for policy interventions to address resource availability within healthcare facilities, particularly in rural areas. Furthermore, exploring ways to improve insurance coverage or develop alternative financing mechanisms for non-insured patients could be beneficial. Future research, potentially qualitative in nature, could delve deeper into patient experiences and explore additional factors influencing satisfaction. Additionally, cost-effectiveness analyses of service quality improvements could be valuable in informing future policy decisions.

### Limitation

This study has some limitations that warrant consideration. One potential limitation is social desirability bias. This refers to the tendency of participants to answer survey questions in a way that they believe will be viewed favorably by the researcher. In the context of our study, participants might have been more likely to report higher satisfaction levels than they truly felt, particularly if they believed such responses would be considered desirable by the healthcare facility. To mitigate this bias, we employed several strategies. First, we ensured that the survey was anonymous and emphasized the importance of honest and truthful responses. Second, the survey questions were phrased in a neutral and objective manner. However, the possibility of social desirability bias cannot be entirely eliminated.

Another limitation arises from the study’s design. The study employed a cross-sectional design, which provides a valuable snapshot of satisfaction levels at a particular point in time. However, it’s important to acknowledge that this design makes it difficult to establish causality. Because the groups weren’t matched and data was collected at one point, we cannot definitively say that any observed differences in satisfaction are solely due to insurance status. Pre-existing differences between insured and uninsured patients may also play a role.

## Conclusion

Overall satisfaction among patients in this study was low, particularly for uninsured patients. Even though there was a significant difference in satisfaction between insured and uninsured patients, both groups reported lower satisfaction compared to studies conducted during the initial rollout of community-based health insurance programs. Notably, despite the short or fair waiting time to be seen, the availability of ordered laboratory tests and prescribed medications within the healthcare facility identified as a key factor influencing patient satisfaction, regardless of insurance status. Having a smaller family size for the insured and residing in an urban area for non-insured patients were positively associated with patient satisfaction.

While this research effectively addressed the main question, it might not capture the full complexity of factors influencing patient satisfaction. Qualitative studies or in-depth interviews could reveal additional insights into patient experiences and perceptions. Therefore, further research is recommended, potentially focusing on qualitative studies to delve deeper into patient experiences and cost-effectiveness analyses of service quality improvements.

### Electronic supplementary material

Below is the link to the electronic supplementary material.

Supplementary Material 1

## Data Availability

Datasets used during the current study is available from the corresponding author on reasonable request through mulu4915@gmail.com.
