# Supplementary material for: Disparities in satisfaction among insured and uninsured adult outpatient department service users in Southern Ethiopia, 2022: a comparative cross-sectional study
Source: BMC Health Serv Res. 2024 Jul 12;24:807. doi: 10.1186/s12913-024-11176-9 (PMC11242004; doi:10.1186/s12913-024-11176-9)
Supplement: Supplementary file 1 — Supplementary Material 1 [file 12913_2024_11176_MOESM1_ESM.docx]

**Annex1: questionnaire**

**Information Sheet English version**

Part one: 1

Full title of the research project: **Differences in Satisfaction among Insured and Uninsured Adult Outpatient Department Service Users in Hadiya Zone Southern Ethiopia**,

Name of the Principal Investigator: Mulugeta Hailu

Name of the organization: Wachemo University

Introduction**:** You have being chosen to participate in this study .I will read the following statements, if you have any questions or anything that is not clear please feel free to ask any time.

**Objective of the study**: the satisfaction levels and factors associated with it among insured and uninsured patients who use outpatient services at Hadiya Zone Southern Ethiopia health facilities were compared.

The information you provide contributes to identifying area where emphasis will be given in developing strategies.

**Participation procedure and guide lines:** The information you provide will be kept anonymous. Your answer completely confidential

**Participant benefit and risk**: Your participation in the study does not involve risk you might feel some mild discomfort from giving your time for answering the questionnaire. But you might experience some benefits from participating in this study.

**Benefit**: You participate in this study does not give any incentives, but you contribute to developing strategies reducing low quality of health care services and financial hardship to health care in our country.

Participant confidentiality**:** All information given by you will be kept strictly confidential. Your name is not written in this form, all information will be store in folder giving code except principal investigator.

**Rights and refuse or withdraw:** You don’t have to answer any question that you don’t want to answer. You are not half or full participating, this study you do not loss any health care services.

Right as participant**:** You have right to ask any question or if you need clarification on the study please Contact: Mulugeta Hailu, Cell phone: 0910101249

Are you willing this study? Yes------date---------------------- No---------date---------------

**Consent form**

**Differences in Satisfaction among Insured and Uninsured Adult Outpatient Department Service Users in Hadiya Zone Southern Ethiopia**

Introduction:

I am __________________________________ I want to carry out this study on Disparities in Satisfaction among Insured and Uninsured Adult Outpatient Department Service Users in Hadiya Zone Southern Ethiopia.

The information I will get from this study will be used in planning and designing intervention to encourage patient to get better quality health care. I am requesting you to participate in this research by giving us the information that we need. You are free to withdraw from the study at any time. But I am urging you to take part till the end to make the study successful.

I have been told of this study and I understand the objectives of the study as the eventual participation in this study is by choice not coercion. I have understood that I am allowed to withdraw from the study any time I feel like and my withdrawal will not affect my right to access to information and health services.

**English version questionnaire**

Name of data collector---------------------------Signature------------Date ----------------
Name of supervisor--------------------------------Signature--------------Date --------------
Questionnaire code------------------------health center/hospital-------------------

| 101 | What is your age? | ---------------------years |
| --- | --- | --- |
| 102 | Sex | Male  Female |
| 103 | What is your marital status? | Married to one spouse  Married to more than one spouse  Not in marriage |
| 104 | Family size | <5  ≥5 |
| 105 | What is your educational status? | Unable to read and write  Grade 1-8  Grade 9 and above |
| 106 | What is your occupation? | Farmer  private  unemployed  student  Other (specify) |
| 107 | Religion | Protestant  Orthodox  Muslim  other |
| 108 | Residency | Urban  Rural |
| 110 | Income | __________ |

**Interactions**

| **201** | interviewed by the language they can understand | 1.YES  2.NO |
| --- | --- | --- |
| **202** | Patients identity is confirmed by service provider | 1.YES  2.NO |
| **203** | Did the provider examine the patient (head to toe) | 1.YES  2.NO |
| **204** | history of past illness | 1.YES  2.NO |
| **205** | history of present illness | 1.YES  2.NO |
| **206** | treatment was taken before arrival at facility | 1.YES  2.NO |
| **207** | provider explain the diagnosis to the patient | 1.YES  2.NO |

**Institutional aspects and patterns of visit among patients**

|  | **Questions** | Response |
| --- | --- | --- |
| **301** | Number of visit within the last 24 months | One time  Two times  Three times  Four time or more |
| **302** | Availability of drinking water | 1 Yes  2. No |
| **303** | Availability of sign and direction | 1 Yes  2. No |
| **304** | Perceived waiting time to see physician | Long  Fair  Short |
| **305** | Perceived consultation duration | Long  Fair  Short |
| **306** | Availability of drinking water | 1 Yes  2. No |
| **307** | Availability of sign and direction | 1. Yes  2. No |
| **308** | Satisfied with availability clean latrine | 1 Satisfied  2 Not satisfied |
| **309** | Laboratory test ordered , if the answer is no skip to Q 206 | 1. Yes  2. No |
| **310** | Availability of ordered tests within the facilities? | All available  Some available  None available |
| **311** | Availability of prescribed drugs | All available  Some available  None available |
| **312** | Information on(dose, frequency, caution and side effects) | All available  Some available  None available |
| **313** | Did the provider explain the diagnosis to the patient? | 1. Yes  2. No |

**Satisfaction Items**

| S.N | Questions | Very  dissatisfied | Dissati  sfied | Neutral | Satisfied | Very  satisfied |
| --- | --- | --- | --- | --- | --- | --- |
| Staff behavior and services | | 1 | 2 | 3 | 4 | 5 |
| 401 | Health providers treats you very friendly and courteous manner | 1 | 2 | 3 | 4 | 5 |
| 402 | Health providers are good to explain how to prevent your disease | 1 | 2 | 3 | 4 | 5 |
| 403 | Health providers are careful to not check everything when treating and examining me | 1 | 2 | 3 | 4 | 5 |
| 404 | You are satisfied with the information provided by health providers (courteous and respectful) | 1 | 2 | 3 | 4 | 5 |
| 405 | You are satisfied with the information provided by all other staffs (other than health providers) | 1 | 2 | 3 | 4 | 5 |
| 406 | You are satisfied with the way health providers listened to you | 1 | 2 | 3 | 4 | 1 |
| 407 | you are satisfied with measures taken to assure your confidentiality | 1 | 2 | 3 | 4 | 5 |
| 408 | you are satisfied with the overall quality of health care services in this health facility | 1 | 2 | 3 | 4 | 5 |
| Physical facilities/environment | | 1 | 2 | 3 | 4 | 5 |
| 409 | Adult OPD location is convenient for you | 1 | 2 | 3 | 4 | 5 |
| 410 | The chairs in the waiting area were confortable | 1 | 2 | 3 | 4 | 5 |
| 411 | Waiting area was not clean | 1 | 2 | 3 | 4 | 5 |
| 412 | Examination/consultation room/Outpatient department was clean | 1 | 2 | 3 | 4 | 5 |
| 413 | Overall the compound is clean | 1 | 2 | 3 | 4 | 5 |
| Accessibility & availability to health care services | | 1 | 2 | 3 | 4 | 5 |
| 414 | Time to get outpatient services after registration (at Waiting area) appropriateness for you | 1 | 2 | 3 | 4 | 5 |
| 415 | You are satisfied with the time spent to get services and get back (overall waiting time) | 1 | 2 | 3 | 4 | 5 |
| 416 | Satisfied with the consultation duration | 1 | 2 | 3 | 4 | 5 |

**Thank you for your genuine response**
